# Effect of venlafaxine on bone loss associated with ligature-induced periodontitis in Wistar rats

**DOI:** 10.1186/1477-5751-9-3

**Published:** 2010-06-14

**Authors:** Rosimary S Carvalho, Carolina M de Souza, Julliana CS Neves, Sergio A Holanda-Pinto, Lívia MS Pinto, Gerly AC Brito, Geanne M de Andrade

**Affiliations:** 1 Laboratory of Neurosciences and Behavior, Department of Physiology and Pharmacology, Federal University of Ceará, Rua Coronel Nunes de Melo, 1127, CEP 60430-270, Fortaleza, CE, Brazil; 2 Department of Clinical Odontology, Faculty of Pharmacy, Odontology and Nursing, Federal University of Ceará, Rua Monsenhor Furtado, s/n, CEP 60441-750, Fortaleza, CE, Brazil; 3 Department of Morphology, Faculty of Medicine, Federal University of Ceará, Rua Delmiro de Farias, s/n, CEP 60416-030, Fortaleza, CE, Brazil

## Abstract

**Background:**

The present study investigated the effects of venlafaxine, an antidepressant drug with immunoregulatory properties on the inflammatory response and bone loss associated with experimental periodontal disease (EPD).

**Materials and Methods:**

Wistar rats were subjected to a ligature placement around the second upper left molar. The treated groups received orally venlafaxine (10 or 50 mg/kg) one hour before the experimental periodontal disease induction and daily for 10 days. Vehicle-treated experimental periodontal disease and a sham-operated (SO) controls were included. Bone loss was analyzed morphometrically and histopathological analysis was based on cell influx, alveolar bone, and cementum integrity. Lipid peroxidation quantification and immunohistochemistry to TNF-α and iNOS were performed.

**Results:**

Experimental periodontal disease rats showed an intense bone loss compared to SO ones (SO = 1.61 ± 1.36; EPD = 4.47 ± 1.98 mm, p < 0.001) and evidenced increased cellular infiltration and immunoreactivity for TNF-α and iNOS. Venlafaxine treatment while at low dose (10 mg/kg) afforded no significant protection against bone loss (3.25 ± 1.26 mm), a high dose (50 mg/kg) caused significantly enhanced bone loss (6.81 ± 3.31 mm, p < 0.05). Venlafaxine effectively decreased the lipid peroxidation but showed no significant change in TNF-α or iNOS immunoreactivity.

**Conclusion:**

The increased bone loss associated with high dose venlafaxine may possibly be a result of synaptic inhibition of serotonin uptake.

## Introduction

Although depression and periodontitis are common conditions in older adults, past studies could not establish that these two conditions are related [1]. However, recent studies evidence that stress and depression may affect the onset and progression of periodontal disease through behavioral and physiologic mechanisms [2,3]. Depression may dysregulate regulatory mechanisms within the brain involved in immune regulation, and thereby alter immune responses and influence the development and progression of infections and inflammatory diseases, including periodontitis [4,5]. In this context, using ligature-induced model of experimental periodontitis Breivik *et al *[6], have shown an enhanced susceptibility to periodontitis in the animal model of depression, which could be reversed by an antidepressant drug, tianeptine. In a clinical situation, depression may thus have a negative effect on periodontal treatment outcome that warrants an antidepressant therapy [7].

Antidepressant treatment contributes to immune regulation in patients with major depressive disorder [8]. Venlafaxine and fluoxetine exert negative immunoregulatory effects by a change in lymphocyte subsets and by suppressing the interferon-γ and interleukin-10 production ratio in whole-blood cells [9,10]. Studies have reported an association between depression and low bone mineral density. Depression may induce bone loss and osteoporotic fractures, primarily via specific immune and endocrine mechanisms, while use of specific antidepressants such as the selective serotonin reuptake inhibitors (SSRIs) are potential contributory factors [11]. Also, there has been a growing body of evidence indicating that inhibition of serotonin uptake has negative effects on the skeleton [12,13]. Venlafaxine is a well-known antidepressant that acts by inhibiting primarily the reuptake of serotonin and noradrenaline [14] and only partially the dopaminergic uptake [15]. Animal studies indicated that it could enhance serotonin and noradrenaline concentrations in hippocampus [16] attenuate anxiety and depression behaviors in REM deprived animals [17] and suppress the central nervous system and peripheral inflammation [18,19].

In the light of these literature findings, the present study was aimed to verify the possible effects of venlafaxine on the inflammatory response and in relation to bone loss associated with ligature-induced experimental periodontal disease in Wistar rats.

## Materials and methods

### Animals

Experiments were performed on male Wistar rats (180-220 g), housed in standard conditions (12-h light/dark cycle and 22 ± 2°C), with free access to food and water except during the test period. The experimental protocol for surgical procedures and animal treatments was duly approved by Institutional Animal Ethics Committee of the Federal University of Ceará in accordance with the guidelines of the National Institute of Health, Bethesda.

### Induction of experimental periodontitis (EPD)

Experimental periodontitis was induced in rats under Ketamine (5%, Vetanarcol^®^, König, Argentina, 60 mg/kg, i.p) - Xylazine (2%, Kensol^®^, König, Argentina, 10 mg/kg, i. p) anesthesia by placement of a sterile nylon (3-0) thread ligature around the cervix of the maxillary left second molar. The ligature was knotted on the buccal side of the tooth, resulting in a subgingival position palatal and a supragingival position buccally, as described elsewhere [20]. The animals were euthanized by cervical dislocation on day 11. The sham group was submitted to the placement and immediate withdrawal of the nylon ligature around the cervix of second upper molar.

### Drug treatments

For treatments, venlafaxine (EFEXOR XR, Wyeth-Whitehall, Brazil) was solubulized in distilled water (vehicle). All treatments (venlafaxine or vehicle) were given orally 1 hr before the induction of EPD, and once daily for 10 days. Animals were assigned randomly to the following six groups. Group 1: sham-operated (SO), Group 2: vehicle-treated experimental periodontitis (EPD); Groups 3 and 4: rats without EPD treated with 10 or 50 mg/kg venlafaxine; Groups 5 and 6: EPD rats treated orally with venlafaxine 10 or 50 mg/kg.

### Measurement of alveolar bone loss

The excised maxillae were fixed in 10% neutral formalin for 24 hours. Both maxillary halves were then defleshed and stained with aqueous methylene blue (1%) in order to differentiate bone from teeth. Measurements of bone loss were made along the axis of each root surfaces of all molar teeth. Three recordings for the first (three roots) and two recordings for the second and third molar teeth (two roots each) were made. The total alveolar bone loss was obtained by taking the sum of the recordings from buccal tooth surface and subtracting the values of the right maxilla (unligated control) from the left one, in millimeters (mm) [21]. Morphometric analysis of the alveolar bone was performed with standardized digital photographed (× 1.5) and the distance was measured with software Image Tool 1.37.

### Histopathological analysis

The alveolar bone specimens were fixed in 10% neutral buffered formalin and demineralized in 5% nitric acid. Following this, these specimens were then dehydrated, embedded in paraffin, and sectioned along the molars in a mesio-distal plane for hematoxylin-eosin. Sections of 6 μm thickness, corresponding, the area between the first and second molars where a ligature had been placed, were evaluated by light microscopy (× 40). Parameters such as inflammatory cell influx, alveolar bone and cementum integrity were analyzed by a histologist in a single-blind fashion and graded as follows: Score 0: absence of or only discrete cellular infiltration (inflammatory cell infiltration is sparse and restricted to the region of the marginal gingival), preserved alveolar process and cementum. Score 1: moderate cellular infiltration (inflammatory cellular infiltration present all over the insert gingival), some but minor alveolar process resorption and intact cementum. Score 2: accentuated cellular infiltration (inflammatory cellular infiltration present in both gingival and periodontal ligament), accentuated degradation of the alveolar process, and partial destruction of cementum. Score 3: accentuated cellular infiltrate, complete resorption of the alveolar process and severe destruction of cementum [22].

### TNF-α and iNOS immunohistochemistry

Thin sections of periodontal tissue (5 μm) were obtained by using a microtome and transferred to a gelatin coated slide. The tissue section was first deparaffinized and then rehydrated. The gingival and periodontal tissues slices after washing with 0.3% Triton X- 100 in phosphate buffer, and quenching of endogenous peroxidase (3% hydrogen peroxide), were incubated with primary antibody (tumor necrosis factor-α (TNF-α), 1:250 or inducible nitric oxide synthase (iNOS), 1:250, Sigma-USA), for overnight at 4°C. After washing with phosphate buffer, the slices were then incubated with secondary antibody for 1 hour, the immunoreactivity to TNF-α was visualized using a colorimetric-based detection kit following the manufacturer protocol (Dako LSAB + Kit, peroxidase, DAKO, USA), and to iNOS using the alkaline phosphatase detection kit (EnVision TM/AP K1396, Dako Cytomation kit).

### Thiobarbituric Acid Reactive Substances (TBARS)

TBARS levels in the gingivomucosal tissue were determined as an indicator of lipid peroxidation according to a previously described method [23]. Gingival tissues were cut into small pieces and then homogenized in ice-cold phosphate buffer (50 mM pH 7.4) to give a 10% homogenate. 250 μL of homogenates were transferred to test tubes and incubated in a water bath at 37°C for 60 min. After this period, 400 μL of 35% perchloric acid was added and centrifuged at 10 500 g for 10 min. To the supernatant solution, 400 μL of 0.6% thiobarbituric acid solution was added and the mixtures were then placed in a water bath and heated for 30 min at 95-100°C. After cooling, the absorbance was measured with a microplate reader at a wavelength of 532 nm. The standard curve was prepared with several concentrations of malondialdehyde (MDA) under the same conditions.

### Statistical Analysis

Data on alveolar bone loss are expressed as mean ± SD. All other data are presented as mean ± S.E.M. Results were analyzed using one-way analysis of variance (ANOVA), followed by Tukey's multiple comparison test. The Kruskal- Wallis and Dunn`s tests were used for histopathological analysis. A significance level of 0.05 was applied.

## Results

### Effect of venlafaxine treatment in EPD

**Figure 1:**
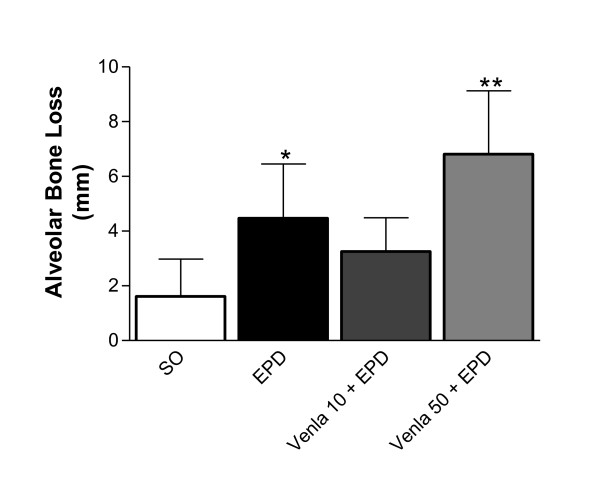
**Effect of venlafaxine treatment on the alveolar bone loss in experimental periodontitis in rats (EPD)**. Measurements were made along the axis of each root of the first molar (three roots) and two recordings for the second and third molar teeth (two roots each). The total alveolar bone loss was obtained by taking the sum of recordings from the buccal tooth and subtracting the value of the right maxilla (unligated control) from the left (mm). Venlafaxine (10 or 50 mg/kg, orally) was administered 1 hour before ligature placement and daily for 10 days. Control groups, sham (SO), and EPD were treated with saline. Values represent mean ± S.D., * vs SO; ** vs EPD (p < 0.05, ANOVA and Tukey's test).

**Figure 2:**
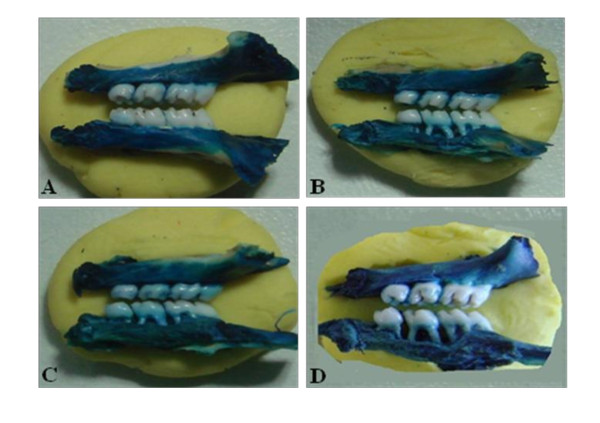
**Macroscopic aspects of periodontium of rats submitted to EPD and treated with venlafaxine**. A sham operated (SO) with no resorption of the alveolar bone when compared to the untreated group (EPD), where severe bone resorption with root exposure is observed B. Figure 2C and 2D shows the periodontium subjected to EPD and treated with venlafaxine (10 or 50 mg/kg, administered orally 1 hour before ligature placement and daily for 10 days) where severe bone loss is observed. Original magnification × 1.5.

Periodontal disease induction by ligature placement caused a significant alveolar bone loss, observed at 11^th ^day (SO = 1.61 ± 1.36 mm; EPD = 4.47 ± 1.98 mm, p < 0.001). Venlafaxine treatment, while at small dose (10 mg/kg) (EPD + Venla 10 = 3.25 ± 1.26 mm) produced no change it caused a significant increase in bone loss at higher dose (50 mg/kg) (EPD + Venla 50 = 6.81 ± 3.31 mm, p < 0.05) (Figure 1). These data can be clearly seen in Figure 2A that shows the macroscopic aspects of the sham group with no resorption of the alveolar bone when compared to the untreated group (EPD), where severe bone resorption with root exposure is observed (Figure 2B). Figures 2C and 2D show the macroscopic appearance of periodontium subjected to experimental periodontitis and treated with venlafaxine 10 or 50 mg/kg, respectively, where severe bone loss is observed.

**Figure 3:**
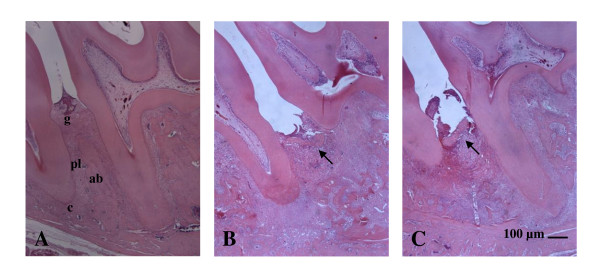
**Histopathology of the periodontium of rats submitted to EPD and treated with venlafaxine**. Venlafaxine (10 mg/kg) was administered orally 1 hour before ligature placement and daily for 10 days). Photomicrographs show the region between the first and second molars of rats: A) sham operated (SO) normal periodontium, where gingival (g), periodontal ligament (pl), alveolar bone (ab), cementum (c), B) periodontium of rat subjected to EPD showing inflammatory cell infiltration with severe cementum destruction and alveolar process, C) periodontium of rat with EPD and treated with venlafaxine showing no evidence of prevention of inflammation or bone resorption (arrow). H&E stain; original magnification × 40. Scale bars = 100 μm.

The histological analysis of the region between the first and second molars of sham operated, shows the structure of the normal periodontium, where gingival (g), periodontal ligament (pl), alveolar bone (ab), cementum (c), can be observed (Figure 3A; Table 1). The histopathology of the periodontium of the animals subjected to experimental periodontitis that received no treatment (EPD) revealed inflammatory cell infiltration coupled with severe cementum destruction and alveolar process destruction (Figure 3B; Table 1), receiving median score 2 (range 2 to 3). The venlafaxine (10 mg/kg) treatment was not able to prevent the inflammatory parameters induced by experimental periodontitis (Figures 3C), receiving median scores 2(1-3) (Table 1). This value was not statistically different when compared to the EPD group. Rats treated with venlafaxine (10 or 50 mg/kg) alone did not manifest bone loss or inflammatory changes in periodontium (not shown in figure 2 or 3).

**Table 1:** Histological analysis of rat maxillae with EPD and treated with venlafaxine.

GROUP	SCORE
SO	0 (0-0)
EPD	2 (2-3) *
EPD + Venlafaxine (10 mg/kg)	2 (1-3) *

Data are reported as medians with range within parenthesis, n = 5. * vs SO (p < 0.004 (Kruskal-Wallis and Dunn' tests).

### Immunohistochemical reaction for TNF- α and iNOS

**Figure 4:**
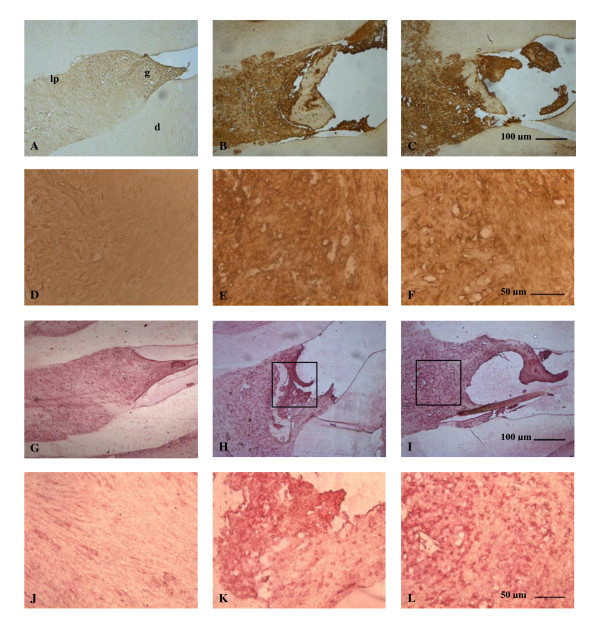
**Photomicrographs of periodontal tissue of rats on EPD and treated with venlafaxine showing the immunoreactivity to TNF-α (A-F) and iNOS (G-L)**. (A,D,G,J) SO: sham operated rats; (B,E,H,K) rats subjected to EPD; (C,F,I,L) rats subjected to EPD and treated with venlafaxine (10 mg/kg). A,B,C,G,H,I -100 ×, bar scale = 100 μm); D,E,F,J,K,L - 400 ×, bar scale = 50 μm). polpa (p) gingival (g), periodontal ligament (pl) and dentina (d).

The periodontium of rats submitted to experimental periodontitis and received no treatment (EPD) showed marked immune-staining for both TNF- α (Figure 4B, 4E) and iNOS (Figure 4H, 4K) when compared to the periodontium of the sham group (Figures 4A, 4D, and 4G, 4J, respectively). Venlafaxine (10 mg/kg) failed to reduce the TNF- α as well as the iNOS immune-staining in the periodontium of rats submitted to experimental periodontitis (Figures 4C, 4F and 4I, 4L, respectively).

### TBARS production in EPD rats and the effect of venlafaxine treatment

**Figure 5:**
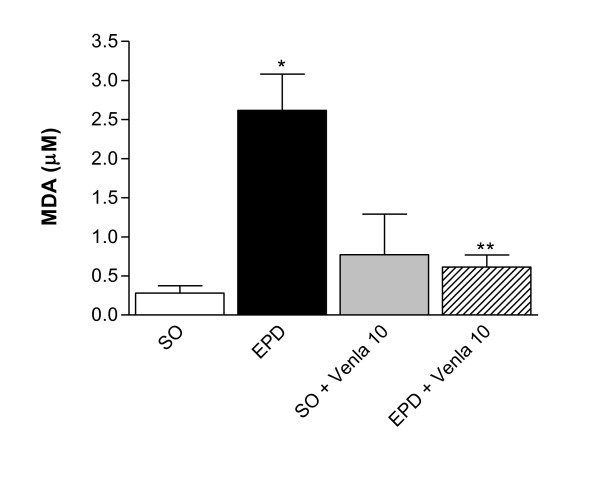
**Malondialdehyde (MDA) levels in gingival tissue of rats submitted to EPD and treated with venlafaxine**. Animals were treated with venlafaxine (10 mg/kg, orally) during 10 days. On day 11, the gingival tissue was removed and analyzed for lipid peroxidation. Values represent mean ± S.E.M. * vs SO, ** vs EPD (p < 0.05, ANOVA and Tukey"s test).

The extent of lipid peroxidation was analyzed in terms of thiobarbituric acid reactive substances (TBARS), expressed as malondialdehyde (MDA) in gingival tissue. Rats submitted to experimental periodontitis (EPD) showed an increase in lipid peroxidation compared with sham group. Venlafaxine (10 mg/kg) prevented (p < 0.05) the malondialdehyde formation (Figure 5).

## Discussion

In the present study, we examined the effects of serotonin-norepinephrine reuptake inhibitor, venlafaxine, on the inflammatory events and bone loss associated with EPD, for three reasons. The first reason is that venlafaxine has been described to possess anti-inflammatory and immunoregulatory effects, and chronic periodontitis is an inflammatory disorder and an immunologically compromised disease [18,19,24]. Secondly, there were few controversial reports on state of depression in patients with periodontitis and depression itself might contribute to bone loss [1,3,25]. A third reason is that there have been reports suggesting that selective serotonin reuptake inhibitors can promote bone loss [12,13]. We used the ligature-induced periodontitis in rats as a model system for the study, since it is a highly reproducible experimental model wherein ligation acts as a mechanical trauma on the dentogingival area, thereby reducing tissue integrity and allowing for intense host-plaque interaction, and finally a bacterial plaque-formation. Lima *et al *have shown that placement of a nylon thread around the second upper molars induced significant alveolar bone loss commencing day-3 of periodontitis induction, reaching a maximum between days 7 and 11, and declining at the 14^th ^day [21]. These data are in accordance with another study that demonstrated a maximum bone loss 9^th ^day after ligature placement, and when sacrificed on day-14, these animals showed shorter but thicker buccal alveolar bone covered by the new bone, and a new dento-epithelial junction formed subjacent to the ligature [20]. The nylon thread functions as a bacterial plaque retentive factor that contributes to periodontitis [26], thus bacterial stimulation induces a host response that leads to inflammatory cell infiltration, osteoclast formation, bone loss, and the loss of tooth attachment [27]. The role of bacteria and the host response in periodontitis has long been recognized. Together, these two factors lead to release of inflammatory mediators and ultimately to alveolar bone loss [28]. Among the mediators, the prostaglandins (PG), mainly PGE2, and the cytokines, interleukin-1 (IL-1) and tumor necrosis factor (TNF-α) play an active role in the development of periodontal inflammation [29]. These cytokines may in turn stimulate nitric oxide (NO) production, which has a role to play in periodontal disease progression and in bone resorption [30,31].

In this study, the periodontium of rats submitted to experimental periodontitis showed marked immunoreactivity to both TNF-α and the iNOS isoenzyme that synthesize NO from L-arginine as compared to periodontium of the SO group, reinforcing the participation of TNF-α and NO in the development of EPD and bone loss. Venlafaxine treatment didn't suppress effectively the TNF-α nor the iNOS immunoreactivities. Vollmar *et al *have shown its immunomodulatory properties, in murine experimental autoimmune encephalomyelitis (EAE), a T-cell-mediated central nervous system demyelinating disease model of multiple sclerosis. Venlafaxine ameliorated the clinical symptoms of the disease possibly by suppressed production of pro-inflammatory cytokines interleukin-12 (IL-12), p40, TNF-α and interferon-γ (IFN-γ). These findings differ from ours, largely due to the reason of differences in the model and treatment protocol, in which venlafaxine was administered at a higher dose (60 mg/kg) and over a longer period (14 days) [32]. Venlafaxine treatment not only failed in preventing the bone loss, but, at a high dose, also significantly enhanced the bone loss. Evidence regarding a functional serotonin (5-hydroxytryptamine) signaling system in bone has generated considerable recent interest. The specific biochemical nature of serotoninergic pathways and their direct and/or indirect effects on bone metabolism are still unclear. Serotonin is involved in the pathophysiology of depression, and therefore studies of depression and antidepressant treatments (as modulators of the serotonin system) are relevant with regard to bone outcomes. SSRIs have been associated with lower bone mineral density (BMD) and increased rates of bone loss, as well as increased rates of fracture after accounting for falls [33]. Selective serotonin-reuptake inhibitors (SSRIs) antagonize the serotonin (5-hydroxytryptamine) transporter (5-HTT), and are frequently prescribed to children and adolescents to treat depression. However, recent findings of functional serotonergic pathways in bone cells and preliminary clinical evidence demonstrating detrimental effects of SSRIs on bone growth have raised questions regarding the effects of these drugs on the growing skeleton. 5-HTT null mutant mice had a consistent skeletal phenotype of reduced mass, altered architecture, and inferior mechanical properties, whereas bone mineral accrual was impaired in growing mice treated with a SSRI [34]. These findings indicate that SSRIs do negatively impact the skeleton and that further research is required to decipher their precise influence.

Our observations on venlafaxine differ from the results obtained in studies of Breivik *et al *[6], in which the use of an antidepressant, tianeptine significantly inhibited the alveolar bone loss in rats on ligature-induced periodontitis. This discrepancy can be clarified the following way. While venlafaxine is a member of SNRIs (serotonin-noradrenalin reuptake inhibitors), tianeptine is an atypical antidepressant drug. In contrast to tricycle antidepressants and selective serotonin reuptake inhibitors (SSRIs), it has been suggested that tianeptine decreases serotonin's bioactivity and its accumulation in serotonergic synapses of the central nervous system by promoting serotonin reuptake, and normalizing serotonergic neurotransmission [35,36]. Venlafaxine, which has a mechanism of action opposite to that of tianeptine (i.e. inhibition of serotonin uptake) clearly explains its dose-related effect on bone loss, observed in the present experiment. Thus we show for the first time that SNRIs such as venlafaxine are likely to worsen the bone loss in periodontal disease.

Oxidative stress has been documented in periodontal disease [37,38]. Patients with periodontitis have a significantly higher level of TBARS than healthy people and this suggests that TBARS of gingival tissue are closely associated with periodontal status and its measurements can help in the treatment and monitoring of progression of periodontal disease [37]. In this study we found that animals submitted to experimental periodontitis had high levels of lipid peroxidation, the finding consistent with earlier observations and the treatment with venlafaxine (10 mg/kg) reduced significantly the lipid peroxidation in these animals. Studies also showed an antioxidant effect of venlafaxine, in rats rendered depressive [39,40]. However, these effects of venlafaxine do not seem to be favorable influenced in this study on the periodontitis outcome (data not shown).

Several lines of evidence suggest that nitric oxide overproduction is associated with periodontal disease, the presence of inducible nitric oxide synthase (iNOS) activity in inflamed gingival tissue of young patients has been demonstrated [41]. Increased iNOS activity has also been reported in rat experimental model of periodontitis, suggesting that the gingivomucosal immune and epithelial cells are able to induce this enzyme [22]. In periodontitis, inducible nitric oxide synthase expression may have beneficial as well as detrimental roles. Beneficial effects may include antimicrobial activity, immune modulation, and inhibition of microvascular thrombosis, as well as increased tissue perfusion. On the other hand, detrimental effects may include a cytotoxic action toward the host tissues, including alveolar bone resorption due to the stimulating effect of nitric oxide on the activity of the osteoclasts [42]. In this study we observed an increase on iNOS immunoreactivity in ligature-induced periodontitis, a finding that corroborates with the study of Lohinai *et al *[30]. It implies that venlafaxine lacks efficacy in suppressing EPD-associated increase in iNOS expression. Venlafaxine also failed to modify the cellular infiltration response in the gingivomucosal tissues, in our experimental conditions.

In conclusion, our results show that the tissue damage induced by ligature is associated with bone loss, inflammatory response and increased immunoreactivity to TNF-α and iNOS. We speculate that the venlafaxine treated rats were not protected against bone loss possibly for the reason that its antidepressant action involves synaptic inhibition of serotonin uptake. Future studies should address on other more selective reuptake inhibitors (SSRIs) to know whether they also behave in same fashion in EPD. Possibly, atypical antidepressants like tianeptine that increase/favour synaptic uptake of serotonin may be more useful to combat periodontitis-associated alveolar bone loss.

## Competing interests

The authors declare that they have no competing interest.

## Authors' contributions

RSC and GMA contributed equally in realizing experiments, data collection and analysis. CMS and JCSN collaborated in immuno-histochemical studies (TNF-α e iNOS) and in the evaluation of oxidant stress. SAHP and LMSP helped in inducing experimental periodontitis in rats. GACB performed the histopathologic analysis. The authors declare that they read and approved the final manuscript.
